# Supplementary material for: A deep learning method for miRNA/isomiR target detection
Source: Sci Rep. 2022 Jun 23;12:10618. doi: 10.1038/s41598-022-14890-8 (PMC9226005; doi:10.1038/s41598-022-14890-8)
Supplement: Supplementary file 1 — Supplementary Information. [file 41598_2022_14890_MOESM1_ESM.docx]

**A Deep Learning Method for MiRNA/IsomiR Target Detection**

Amlan Talukder, Wencai Zhang, Xiaoman Li and Haiyan Hu

| **Table S1:** Samples, reads and aligned chimeric reads for CLASH and CLEAR-CLIP data | | | | | |
| --- | --- | --- | --- | --- | --- |
|  | Sample | Run | # Reads | # Aligned Chimeric Reads  (Alignments in minus strand and with gaps filtered) | # Aligned Chimeric Reads  (Alignment pairs with more than 4 nt gap or overlap filtered) |
| CLASH | E1_A12 | SRR959751.2 | 5304756 | 64603 | 9821 |
|  | E2_0727 | SRR959752.2 | 1194099 | 34454 | 6063 |
|  | E2_0727HS | SRR959753.1 | 9398823 | 78246 | 11115 |
|  | E3_L1 | SRR959754.2 | 1304661 | 45637 | 11201 |
|  | E3_L1HS | SRR959755.1 | 7216467 | 77193 | 20359 |
|  | E4_L2 | SRR959756.2 | 2598477 | 100448 | 24793 |
|  | E4_L2HS | SRR959757.1 | 16428827 | 213935 | 68545 |
|  | E5_TA1 | SRR959758.2 | 6629002 | 86710 | 11174 |
|  | E6_TAON | SRR959759.2 | 5600597 | 70655 | 10360 |
| CLEAR-CLIP | 1 | SRR2413175.1 | 8795072 | 42596 | 7672 |
|  | 2 | SRR2413176.1 | 2384028 | 12303 | 2963 |
|  | 3 | SRR2413177.1 | 4686830 | 7750 | 676 |
|  | 4 | SRR2413178.1 | 6794961 | 33624 | 7540 |
|  | 5 | SRR2413179.1 | 1699691 | 25244 | 10836 |
|  | 6 | SRR2413180.1 | 977323 | 13738 | 4184 |
|  | 7 | SRR2413181.1 | 1656605 | 17950 | 3272 |
|  | 8 | SRR2413182.1 | 859801 | 14069 | 2277 |
|  | 9 | SRR2413183.1 | 2619165 | 34505 | 5272 |
|  | 10 | SRR2413184.1 | 1552306 | 17394 | 2303 |
|  | 11 | SRR2413185.1 | 1433937 | 21114 | 3647 |
|  | 12 | SRR2413186.1 | 1097284 | 14426 | 2396 |

**Table S2:** Number of exact miRNAs, different types of isomiRs, targets, interactions and reads in the positive and negative interactions of CLASH training, test, CLEAR-CLIP and miRTarBase datasets. The number inside the parenthesis represents the number of miRNAs that produced the corresponding isomiRs.

|  | EXACT | 5' | 3' | Polymorphic | # Total isomiRs | # Targets | # Interactions | # Reads |
| --- | --- | --- | --- | --- | --- | --- | --- | --- |
| CLASH (pos) | 122 | 447 (99) | 957 (188) | 281 (61) | 1104 (217) | 10415 | 70213 | 70213 |
| CLASH (neg) | 120 | 332 (78) | 814 (169) | 192 (47) | 931 (197) | 5254 | 41646 | NA |
| CLASH Training (pos) | 122 | 447 (99) | 957 (188) | 281 (61) | 1104 (217) | 9713 | 56160 | 56160 |
| CLASH Training (neg) | 119 | 325 (77) | 800 (166) | 185 (46) | 914 (196) | 4988 | 33327 | NA |
| CLASH Test (pos) | 121 | 423 (93) | 921 (186) | 265 (57) | 1056 (213) | 5004 | 14053 | 14053 |
| CLASH Test (neg) | 113 | 263 (66) | 657 (144) | 147 (36) | 751 (177) | 3029 | 8319 | NA |
| CLEAR-CLIP (pos) | 0 | 691 (161) | 429 (142) | 205 (64) | 764 (170) | 975 | 14684 | 14684 |
| CLEAR-CLIP (neg) | 0 | 270 (92) | 180 (79) | 108 (39) | 295 (99) | 360 | 1323 | NA |
| mirTarBase | 573 | NA | NA | NA | NA | 5277 | 14144 | NA |

**Table S3:** Number of eight types of isomiRs occurring in CLASH and CLEAR-CLIP datasets. The isomiRs representing multiple types are shown under “Hybrid” column.

|  | 5' add | 5' del | 5' rep | 3' add | 3' del | 3' rep | SNP | MNP | Hybrid |
| --- | --- | --- | --- | --- | --- | --- | --- | --- | --- |
| CLASH | 20 | 39 | 9 | 361 | 96 | 92 | 12 | 11 | 464 |
| CLEAR-CLIP | 259 | 0 | 14 | 14 | 1 | 7 | 0 | 0 | 469 |

| **Table S4:** 10-fold cross validation on the 80% training data | | | | | | | | | |
| --- | --- | --- | --- | --- | --- | --- | --- | --- | --- |
| Pos | Neg | AUROC | AUPR | F1 | MCC | Accuracy | Precision | Recall | Specificity |
| 5616 | 3333 | 0.9952 | 0.9973 | 0.9592 | 0.8880 | 0.9473 | 0.9311 | 0.9891 | 0.8767 |
| 5616 | 3333 | 0.9952 | 0.9972 | 0.9639 | 0.9012 | 0.9534 | 0.9373 | 0.9922 | 0.8881 |
| 5616 | 3333 | 0.9933 | 0.9963 | 0.9574 | 0.8828 | 0.9449 | 0.9297 | 0.9868 | 0.8743 |
| 5616 | 3333 | 0.9950 | 0.9971 | 0.9589 | 0.8871 | 0.9466 | 0.9275 | 0.9925 | 0.8692 |
| 5616 | 3333 | 0.9938 | 0.9966 | 0.9588 | 0.8868 | 0.9468 | 0.9321 | 0.9872 | 0.8788 |
| 5616 | 3333 | 0.9945 | 0.9968 | 0.9590 | 0.8874 | 0.9468 | 0.9285 | 0.9916 | 0.8713 |
| 5616 | 3333 | 0.9951 | 0.9973 | 0.9617 | 0.8948 | 0.9504 | 0.9335 | 0.9916 | 0.8809 |
| 5616 | 3332 | 0.9947 | 0.9970 | 0.9599 | 0.8899 | 0.9481 | 0.9316 | 0.9900 | 0.8776 |
| 5616 | 3332 | 0.9939 | 0.9966 | 0.9555 | 0.8774 | 0.9422 | 0.9246 | 0.9886 | 0.8640 |
| 5616 | 3332 | 0.9941 | 0.9967 | 0.9601 | 0.8903 | 0.9485 | 0.9340 | 0.9877 | 0.8824 |

**Table S5:** Performance comparison on the interactions involving different types of isomiRs in the CLASH 20% test data and CLEAR-CLIP data.

|  | IsomiR Types | Pos | Neg | AUROC | AUPR | F1 | MCC | Accuracy | Precision | Recall | Specificity |
| --- | --- | --- | --- | --- | --- | --- | --- | --- | --- | --- | --- |
| CLASH test | EXACT | 2414 | 2065 | 0.9847 | 0.9879 | 0.9133 | 0.8057 | 0.9002 | 0.8591 | 0.9747 | 0.8131 |
|  | 5' | 3789 | 1086 | 0.9960 | 0.9989 | 0.9732 | 0.8745 | 0.9573 | 0.9520 | 0.9952 | 0.8250 |
|  | 3' | 9603 | 5784 | 0.9958 | 0.9976 | 0.9645 | 0.9036 | 0.9544 | 0.9385 | 0.9919 | 0.8921 |
|  | Polymorphic | 3131 | 737 | 0.9949 | 0.9988 | 0.9756 | 0.8657 | 0.9597 | 0.9564 | 0.9955 | 0.8073 |
| CLEAR-CLIP | EXACT | 0 | 0 | NA | NA | NA | NA | NA | NA | NA | NA |
|  | 5' | 13180 | 1225 | 0.9369 | 0.9937 | 0.9344 | 0.5393 | 0.8857 | 0.9836 | 0.8899 | 0.8400 |
|  | 3' | 8485 | 828 | 0.9418 | 0.9941 | 0.9439 | 0.5848 | 0.9018 | 0.9836 | 0.9072 | 0.8454 |
|  | Polymorphic | 4317 | 426 | 0.9167 | 0.9917 | 0.9136 | 0.4975 | 0.8533 | 0.9837 | 0.8529 | 0.8568 |

**Table S6:** Performance comparison between DMISO with the five external tools on the target sites of miRNAs and isomiRs in CLASH 20% test data.

|  |  | Pos | Neg | AUROC | AUPR | F1 | MCC | Accuracy | Precision | Recall | Specificity |
| --- | --- | --- | --- | --- | --- | --- | --- | --- | --- | --- | --- |
| miRNAs | DMISO | 2414 | 2065 | 0.9847 | 0.9879 | 0.9133 | 0.8057 | 0.9002 | 0.8591 | 0.9747 | 0.8131 |
|  | miRanda | 2414 | 2065 | 0.6387 | 0.6652 | 0.4383 | 0.3832 | 0.6109 | 0.9869 | 0.2817 | 0.9956 |
|  | RNA22 | 2414 | 2065 | 0.4998 | 0.5390 | None | -0.0162 | 0.4608 | 0.0000 | 0.0000 | 0.9995 |
|  | TargetScan | 2414 | 2065 | 0.5993 | 0.6271 | 0.3425 | 0.3084 | 0.5689 | 0.9618 | 0.2084 | 0.9903 |
|  | miRAW | 2414 | 2065 | 0.6083 | 0.6023 | 0.6490 | 0.2177 | 0.6126 | 0.6342 | 0.6645 | 0.5521 |
|  | miTAR | 2414 | 2065 | 0.6247 | 0.6223 | 0.5547 | 0.2636 | 0.6111 | 0.7243 | 0.4495 | 0.8000 |
| IsomiRs | DMISO | 11639 | 6254 | 0.9928 | 0.9964 | 0.9691 | 0.9134 | 0.9602 | 0.9776 | 0.9608 | 0.9591 |
|  | miRanda | 11639 | 6254 | 0.5766 | 0.7032 | 0.2722 | 0.2391 | 0.4506 | 0.9845 | 0.1579 | 0.9954 |
|  | RNA22 | 11639 | 6254 | 0.5003 | 0.6506 | 0.0027 | 0.0080 | 0.3501 | 0.7619 | 0.0014 | 0.9992 |
|  | TargetScan | 11639 | 6254 | 0.5480 | 0.6825 | 0.1899 | 0.1772 | 0.4148 | 0.9541 | 0.1054 | 0.9906 |
|  | miRAW | 11639 | 6254 | 0.6174 | 0.7127 | 0.6567 | 0.2239 | 0.6059 | 0.7578 | 0.5793 | 0.6554 |
|  | miTAR | 11639 | 6254 | 0.5905 | 0.6994 | 0.5245 | 0.1830 | 0.5322 | 0.7739 | 0.3967 | 0.7843 |

**Table S7:** Common pathways of the miRNA clusters in the CLASH 20% test data and CLEAR-CLIP data.

|  | Cluster | Number of common Targets | Common KEGG pathway |
| --- | --- | --- | --- |
| CLASH | hsa-miR-106a-5p, hsa-miR-20a-5p, hsa-miR-20b-5p, hsa-miR-26b-5p, hsa-miR-93-5p | 194 | Hepatitis B |
|  | hsa-miR-106a-5p, hsa-miR-17-5p, hsa-miR-20a-5p, hsa-miR-20b-5p, hsa-miR-26b-5p | 194 | Hepatitis B |
|  | hsa-miR-106a-5p, hsa-miR-17-5p, hsa-miR-196a-5p, hsa-miR-196b-5p, hsa-miR-20b-5p | 81 | Proteoglycans in cancer, TGF-beta signaling pathway, Hepatitis B, FoxO signaling pathway, Chronic myeloid leukemia |
|  | hsa-miR-106a-5p, hsa-miR-20a-5p, hsa-miR-20b-5p, hsa-miR-421 | 73 | TGF-beta signaling pathway, Lysine degradation |
|  | hsa-miR-181b-5p, hsa-miR-196a-5p, hsa-miR-196b-5p, hsa-miR-20b-5p | 33 | Proteoglycans in cancer, p53 signaling pathway |
|  | hsa-miR-181b-5p, hsa-miR-20a-5p, hsa-miR-20b-5p, hsa-miR-421 | 32 | p53 signaling pathway |
|  | hsa-let-7a-5p, hsa-let-7d-5p, hsa-miR-4516 | 4 | Lysine degradation |
|  | hsa-let-7d-5p, hsa-let-7g-5p, hsa-let-7i-5p | 807 | Cell cycle, Hippo signaling pathway, Viral carcinogenesis, Proteoglycans in cancer, Hepatitis B |
| CLEAR-CLIP | hsa-miR-106a-5p, hsa-miR-1299, hsa-miR-20a-5p, hsa-miR-20b-5p | 11 | Hepatitis B |
|  | hsa-miR-17-5p, hsa-miR-20a-5p, hsa-miR-20b-5p, hsa-miR-93-5p | 687 | Hepatitis B, Pathways in cancer, Proteoglycans in cancer, Chronic myeloid leukemia, FoxO signaling pathway |
|  | hsa-let-7a-5p, hsa-miR-1268a, hsa-miR-1277-5p | 1 | Pathways in cancer, Transcriptional misregulation in cancer |
|  | hsa-miR-320b, hsa-miR-320c, hsa-miR-320d | 513 | Hippo signaling pathway, Adherens junction |
|  | hsa-let-7a-5p, hsa-miR-1268a | 9 | Lysine degradation, Viral carcinogenesis, Bacterial invasion of epithelial cells, Chronic myeloid leukemia, Pathways in cancer, Transcriptional misregulation in cancer |
|  | hsa-miR-1268a, hsa-miR-1268b | 5 | Lysine degradation, Viral carcinogenesis, |
|  | hsa-miR-1277-5p, hsa-miR-138-5p | 27 | Pathways in cancer, Proteoglycans in cancer |

**Table S8:** Performance comparison between DMISO, trained on the target sites of 109 out of total 217 CLASH miRNAs, with the five external tools on the target sites of rest of the 108 CLASH miRNAs.

|  | Pos | Neg | AUROC | AUPR | F1 | MCC | Accuracy | Precision | Recall | Specificity |
| --- | --- | --- | --- | --- | --- | --- | --- | --- | --- | --- |
| DMISO | 7266 | 5429 | 0.9889 | 0.9923 | 0.9547 | 0.8954 | 0.9486 | 0.9630 | 0.9465 | 0.9514 |
| miRanda | 7266 | 5429 | 0.5998 | 0.6567 | 0.3365 | 0.3068 | 0.5423 | 0.9886 | 0.2027 | 0.9969 |
| RNA22 | 7266 | 5429 | 0.5006 | 0.5728 | 0.0027 | 0.0200 | 0.4284 | 0.9091 | 0.0014 | 0.9998 |
| TargetScan | 7266 | 5429 | 0.5777 | 0.6369 | 0.2778 | 0.2616 | 0.5176 | 0.9703 | 0.1621 | 0.9934 |
| miRAW | 7266 | 5429 | 0.6122 | 0.6395 | 0.6014 | 0.2241 | 0.5999 | 0.6997 | 0.5274 | 0.6970 |
| miTAR | 7266 | 5429 | 0.6073 | 0.6391 | 0.5413 | 0.2242 | 0.5818 | 0.7271 | 0.4312 | 0.7834 |

**Table S9:** Performance comparison between DMISO trained on top 5 pairs of miRNA/isomiR-mRNA target sites with external tools on CLASH 20% test, CLEAR-CLIP and miRTarBase datasets.

|  |  | Pos | Neg | AUROC | AUPR | F1 | MCC | Accuracy | Precision | Recall | Specificity |
| --- | --- | --- | --- | --- | --- | --- | --- | --- | --- | --- | --- |
| CLASH test | DMISO | 42561 | 25804 | 0.9942 | 0.9966 | 0.9667 | 0.9102 | 0.9579 | 0.9512 | 0.9827 | 0.9169 |
|  | miRanda | 42561 | 25804 | 0.6022 | 0.6987 | 0.3450 | 0.2929 | 0.5058 | 0.9869 | 0.2090 | 0.9954 |
|  | RNA22 | 42561 | 25804 | 0.5006 | 0.6229 | 0.0033 | 0.0155 | 0.3783 | 0.8353 | 0.0017 | 0.9995 |
|  | TargetScan | 42561 | 25804 | 0.5647 | 0.6695 | 0.2424 | 0.2195 | 0.4603 | 0.9612 | 0.1387 | 0.9908 |
|  | miRAW | 42561 | 25804 | 0.5497 | 0.6531 | 0.2678 | 0.1469 | 0.4543 | 0.8129 | 0.1603 | 0.9392 |
|  | miTAR | 42561 | 25804 | 0.5946 | 0.6779 | 0.4939 | 0.2018 | 0.5376 | 0.7753 | 0.3624 | 0.8268 |
| CLEAR-CLIP | DMISO | 45752 | 4388 | 0.9559 | 0.9954 | 0.9332 | 0.5699 | 0.8846 | 0.9894 | 0.8830 | 0.9011 |
|  | miRanda | 45752 | 4388 | 0.5285 | 0.9174 | 0.1414 | 0.0627 | 0.1554 | 0.9765 | 0.0762 | 0.9809 |
|  | RNA22 | 45752 | 4388 | 0.4996 | 0.9124 | 0.0010 | -0.0102 | 0.0879 | 0.7931 | 0.0005 | 0.9986 |
|  | TargetScan | 45752 | 4388 | 0.6124 | 0.9320 | 0.3967 | 0.1513 | 0.3118 | 0.9911 | 0.2480 | 0.9768 |
|  | miRAW | 45752 | 4388 | 0.5019 | 0.9128 | 0.1661 | 0.0038 | 0.1632 | 0.9158 | 0.0913 | 0.9125 |
|  | miTAR | 45752 | 4388 | 0.6756 | 0.9425 | 0.6294 | 0.2003 | 0.5012 | 0.9772 | 0.4642 | 0.8870 |
| miRTarBase | DMISO | 14144 | 0 | NA | NA | NA | NA | NA | NA | 0.8249 | NA |
|  | miRanda | 14144 | 0 | NA | NA | NA | NA | NA | NA | 0.7045 | NA |
|  | RNA22 | 14144 | 0 | NA | NA | NA | NA | NA | NA | 0.0199 | NA |
|  | TargetScan | 14144 | 0 | NA | NA | NA | NA | NA | NA | 0.7632 | NA |
|  | miRAW | 14144 | 0 | NA | NA | NA | NA | NA | NA | 0.6734 | NA |
|  | miTAR | 14144 | 0 | NA | NA | NA | NA | NA | NA | 0.0090 | NA |


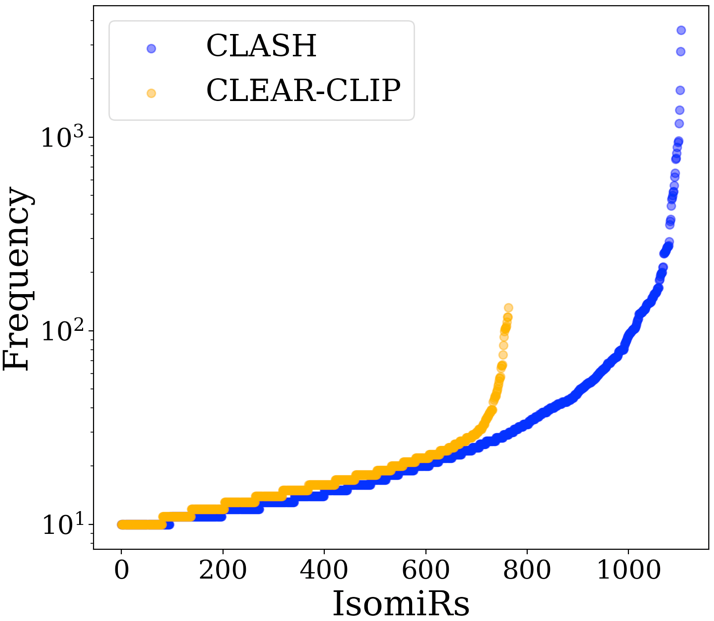


**Figure S1:** Frequencies of the 1,104 and 764 isomiRs in the CLASH and CLEAR-CLIP datasets respectively. The Y-axis is shown in log scale.


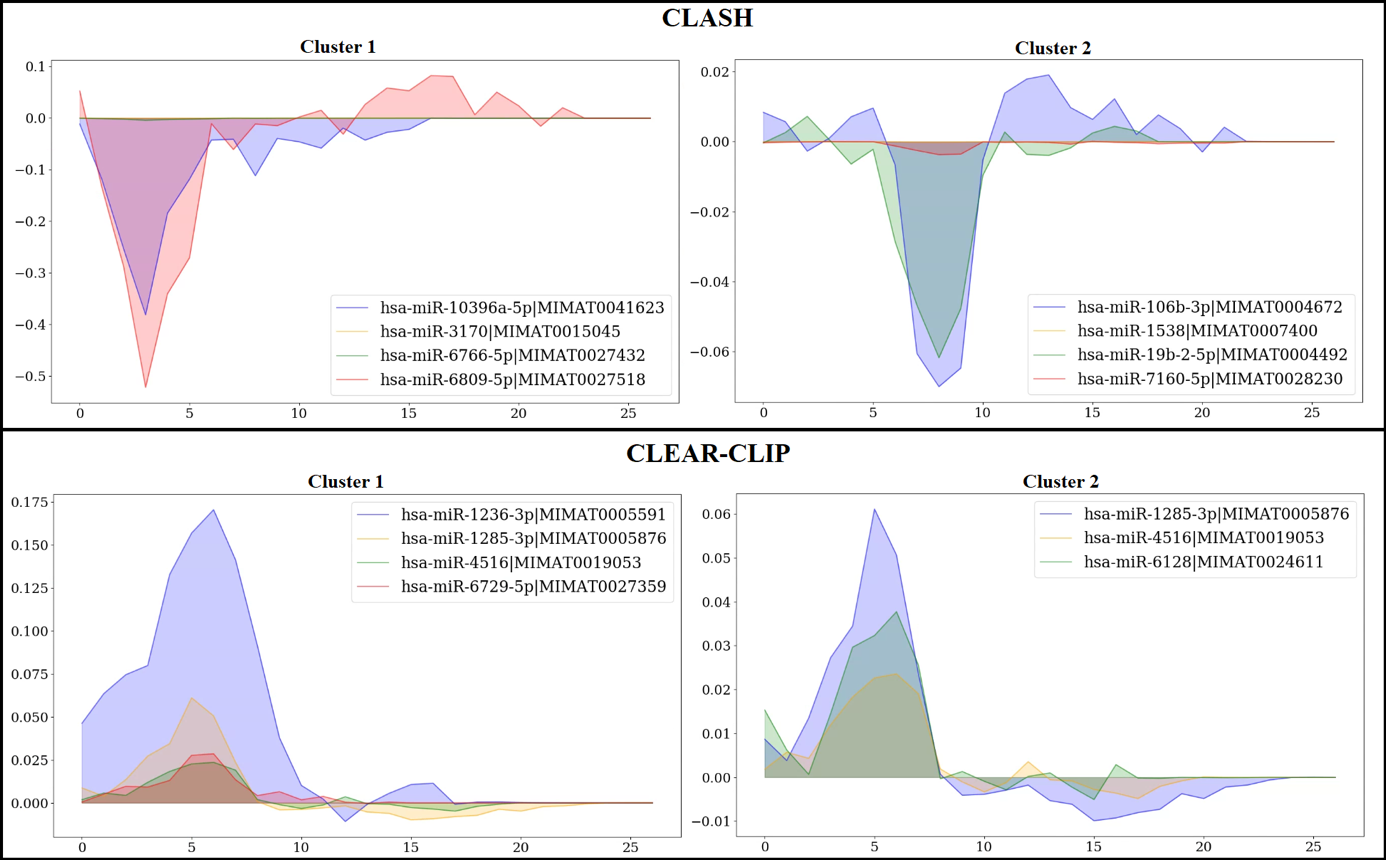


**Figure S2:** The cluster of miRNAs for which DMISO reacted similarly to the position-wise changes. The top 2 clusters of miRNAs are shown for the two datasets. The X-axis represents the miRNA positions and Y-axis represents the changes in DMISO’s prediction based on the changes in corresponding miRNA positions.
